# Phase Alignment of Low-Frequency Neural Activity to the Amplitude Envelope of Speech Reflects Evoked Responses to Acoustic Edges, Not Oscillatory Entrainment

**DOI:** 10.1523/JNEUROSCI.1663-22.2023

**Published:** 2023-05-24

**Authors:** Yulia Oganian, Katsuaki Kojima, Assaf Breska, Chang Cai, Anne Findlay, Edward Chang, Srikantan S. Nagarajan

**Affiliations:** ^1^Department of Neurological Surgery, University of California–San Francisco, San Francisco, California 94158; ^2^Center for Integrative Neuroscience, University Medical Center Tuebingen, Tuebingen 72076, Germany; ^3^Department of Radiology, University of California–San Francisco, San Francisco, California 94143-0628; ^4^Department of Pediatrics, University of California, San Francisco, Box 0734, 550 16th Street, 5th Floor, San Francisco, CA 94158, USA; ^5^Neurodevelomental Disorders Prevention Center, Perinatal Institute, Cincinnati Children's Hospital Medical Center, Cincinnati, Ohio 45229-3039; ^6^Department of Pediatrics, University of Cincinnati College of Medicine, 3230 Eden Avenue, Cincinnati, OH 45267; ^7^Max-Planck-Institute for biological Cybernetics, Tuebingen 72076, Germany

**Keywords:** evoked response, language, MEG, modeling, neural oscillations, speech

## Abstract

The amplitude envelope of speech is crucial for accurate comprehension. Considered a key stage in speech processing, the phase of neural activity in the theta-delta bands (1-10 Hz) tracks the phase of the speech amplitude envelope during listening. However, the mechanisms underlying this envelope representation have been heavily debated. A dominant model posits that envelope tracking reflects entrainment of endogenous low-frequency oscillations to the speech envelope. Alternatively, envelope tracking reflects a series of evoked responses to acoustic landmarks within the envelope. It has proven challenging to distinguish these two mechanisms. To address this, we recorded MEG while participants (*n* = 12, 6 female) listened to natural speech, and compared the neural phase patterns to the predictions of two computational models: an oscillatory entrainment model and a model of evoked responses to peaks in the rate of envelope change. Critically, we also presented speech at slowed rates, where the spectro-temporal predictions of the two models diverge. Our analyses revealed transient theta phase-locking in regular speech, as predicted by both models. However, for slow speech, we found transient theta and delta phase-locking, a pattern that was fully compatible with the evoked response model but could not be explained by the oscillatory entrainment model. Furthermore, encoding of acoustic edge magnitudes was invariant to contextual speech rate, demonstrating speech rate normalization of acoustic edge representations. Together, our results suggest that neural phase-locking to the speech envelope is more likely to reflect discrete representation of transient information rather than oscillatory entrainment.

**SIGNIFICANCE STATEMENT** This study probes a highly debated topic in speech perception: the neural mechanisms underlying the cortical representation of the temporal envelope of speech. It is well established that the slow intensity profile of the speech signal, its envelope, elicits a robust brain response that “tracks” these envelope fluctuations. The oscillatory entrainment model posits that envelope tracking reflects phase alignment of endogenous neural oscillations. Here the authors provide evidence for a distinct mechanism. They show that neural speech envelope tracking arises from transient evoked neural responses to rapid increases in the speech envelope. Explicit computational modeling provides direct and compelling evidence that evoked responses are the primary mechanism underlying cortical speech envelope representations, with no evidence for oscillatory entrainment.

## Introduction

Speech comprehension is essential to human communication. A major computational step in neural processing of speech is the extraction of its amplitude envelope, the overall intensity of speech across spectral bands. The speech envelope is dominated by fluctuations in the range of ∼1-10 Hz, which are temporally correlated with the syllabic structure of speech, and the removal of which from speech severely impairs intelligibility (Drullman et al., 1994a,b). Many studies have shown a consistent relationship between the phase of band-limited low-frequency neural activity measured in MEG/EEG over auditory cortical areas and the phase of the amplitude envelope of speech, a phenomenon widely known as envelope tracking (Ahissar et al., 2001; Luo and Poeppel, 2007). The strength of envelope tracking is correlated with speech intelligibility, suggesting that it could constitute an essential stage in speech comprehension (Abrams et al., 2008; Peelle et al., 2013). However, the neural computations underlying speech envelope tracking are controversial (Zoefel et al., 2018b; Obleser and Kayser, 2019; Gwilliams, 2020).

A dominant theory of speech envelope tracking posits that it reflects the entrainment (i.e., phase alignment) of endogenous neural oscillations to envelope fluctuations. According to this, phase correction is driven by discrete acoustic landmarks in the speech signal and occurs primarily for oscillators in the theta-delta range (1-10 Hz), matching the syllabic rate of the speech signal (Giraud and Poeppel, 2012; Ding et al., 2016; Zoefel, 2018). Functionally, oscillatory entrainment is thought to benefit speech processing via the self-sustaining property of oscillating dynamical systems, resulting in automatically driven temporal prediction of upcoming information (Haegens and Zion Golumbic, 2018; Helfrich et al., 2019; Hovsepyan et al., 2020).

However, recent work has demonstrated that phase alignment of low-frequency neural activity can be the outcome of transient neural responses rather than oscillatory dynamics (Capilla et al., 2011; Breska and Deouell, 2017). This becomes pertinent in the case of speech, as it has been suggested that the speech envelope is encoded in evoked responses to the same acoustic landmarks that supposedly drive the entrainment process. Recent electrophysiology recordings suggest that these events are peaks in the rate of amplitude envelope change, marking the perceived onset of vowels. To date, it remains unclear which of these processes drive phase adjustments in speech envelope tracking. The two competing models have drastically disparate functional and mechanistic implications (Ruhnau et al., 2020; Zoefel et al., 2020; Bree et al., 2021; Doelling and Assaneo, 2021).

To address this, we combined a model-based computational approach with neurophysiological (MEG) recordings of neural responses in an ecologically valid context, using natural continuous speech. We implemented an oscillatory entrainment model and an evoked responses model, quantified the spectral content and temporal dynamics of neural activity predicted by each model in response to speech, identified diverging model predictions, and tested them against MEG data.

Our modeling approach had two critical features. First, we analyzed phase patterns as event-locked to acoustic landmarks. This allowed us to have an extremely high number of events (2106 within-participant), and to probe phase alignment in a time-resolved manner. Particularly, it enabled us to quantify reverberation following a phase reset, a hallmark of oscillatory processes. Second, we additionally presented continuous speech at, equally intelligible, one-third of its original rate. In natural speech, the speech rate, and hence the expected frequency of an entrained oscillator, overlaps with the spectral content of evoked responses. Moreover, the duration of an evoked response is longer than the time between phase resetting events, where oscillatory reverberation is expected to occur. We hypothesized that slowing speech would solve both.

This manipulation also allowed us to address the neural mechanisms of speech rate normalization, listeners' ability to adjust perceptual processes to differences in speech rate. It has previously been proposed that speech rate normalization relies on shifts in the frequency of the phase-locked oscillator toward the speech rate (Nourski et al., 2009; Pefkou et al., 2017; Kösem et al., 2018). Here we examined this hypothesis in naturalistic speech.

## Materials and Methods

### Participants

Twelve healthy, right-handed volunteers (6 females; age range 22-44 years, median 25 years) participated in the study. All participants were native speakers of English. All participants provided informed written consent and received monetary compensation for their participation. The study was approved by the University of California, San Francisco Committee on Human Research.

### Speech stimulus

Participants listened to two stories (one male, one female speaker) from the Boston University Radio Speech Corpus (for full stimulus transcripts, see Extended Data Table 1-1) (Ostendorf et al., 1995), each once at regular speech rate and once slowed to one-third speech rate. Overall, the stimuli contained 26 paragraphs (each containing 1-4 sentences) of 10-60 s duration, with silent periods of 500-1100 ms inserted between paragraphs to allow measuring onset responses in the MEG without distortion from preceding speech. Boundaries between paragraphs corresponded to breaks between phrases, such that silences were perceived as natural. Speech stimuli were slowed using the Pitch Synchronous Overlap and Add algorithm, as implemented in the software Praat (Boersma and Weenik, 2019), which slows down the temporal structure of the speech signal while keeping its spectral structure constant (Moulines and Charpentier, 1990). Overall, the regular speech stimulus was 6.5 min long and the slowed stimulus was 19.5 min long. An example excerpt of the stimulus at slow and regular speech rate is provided in Extended Data 1 and 2.

10.1523/JNEUROSCI.1663-22.2023.t1-1 Table 1-1 Speech stimulus transcription. Download Table 1-1, DOCX file.

10.1523/JNEUROSCI.1663-22.2023.ext1 Extended Data 1 Sound files for an example stimulus at regular and slowed speech rates. Download Extended Data 1, WAV file.

10.1523/JNEUROSCI.1663-22.2023.ext2 Extended Data 2 Annotation of content of Sound 1-1 in a praat textgrid format. Download Extended Data 2, TXT file.

### Procedure and stimulus presentation

All stimuli were presented binaurally at a comfortable ambient loudness (∼70 dB) through MEG-compatible headphones using custom-written MATLAB R2012b scripts (The MathWorks, https://www.mathworks.com). Speech stimuli were sampled at 16 kHz. Participants were asked to listen to the stimuli attentively and to keep their eyes closed throughout.

Participants listened to the radio stories once at regular and once at slowed rate in separate but interleaved blocks, such that each participant heard one story first at regular speech rate and the other at slowed speech rate. Comprehension was assessed with 3 or 4 multiple choice comprehension questions posed after each story (for list of comprehension questions, see Extended Data Table 1-2). For each participant, a different randomly selected subset of questions was used for each block. Percentage correct was compared between regular and slow blocks using a two-sided paired *t* test.

10.1523/JNEUROSCI.1663-22.2023.t1-2 Table 1-2 Comprehension questions. Download Table 1-2, DOCX file.

### Neural data acquisition and preprocessing

MEG recordings were obtained with a 275-axial gradiometers whole-head MEG system (CTF) at a sampling rate of 1200 Hz. Three fiducial coils were placed on the nasion and left and right pre-auricular points to triangulate the position of the head relative to the MEG sensor array. The position of the patient's head in the device relative to the MEG sensors was determined using indicator coils before and after each recording interval to verify an adequate sampling of the entire field. The fiducial markers were later coregistered onto a structural MRI scan to generate head shape (Teichmann et al., 2013).

### Data analysis and modeling

All analyses were conducted in MATLAB R2019a-MATLAB R2021b (The MathWorks, https://www.mathworks.com) using custom-written scripts and the FieldTrip toolbox (Oostenveld et al., 2011).

### Acoustic feature extraction

**Figure 1.**
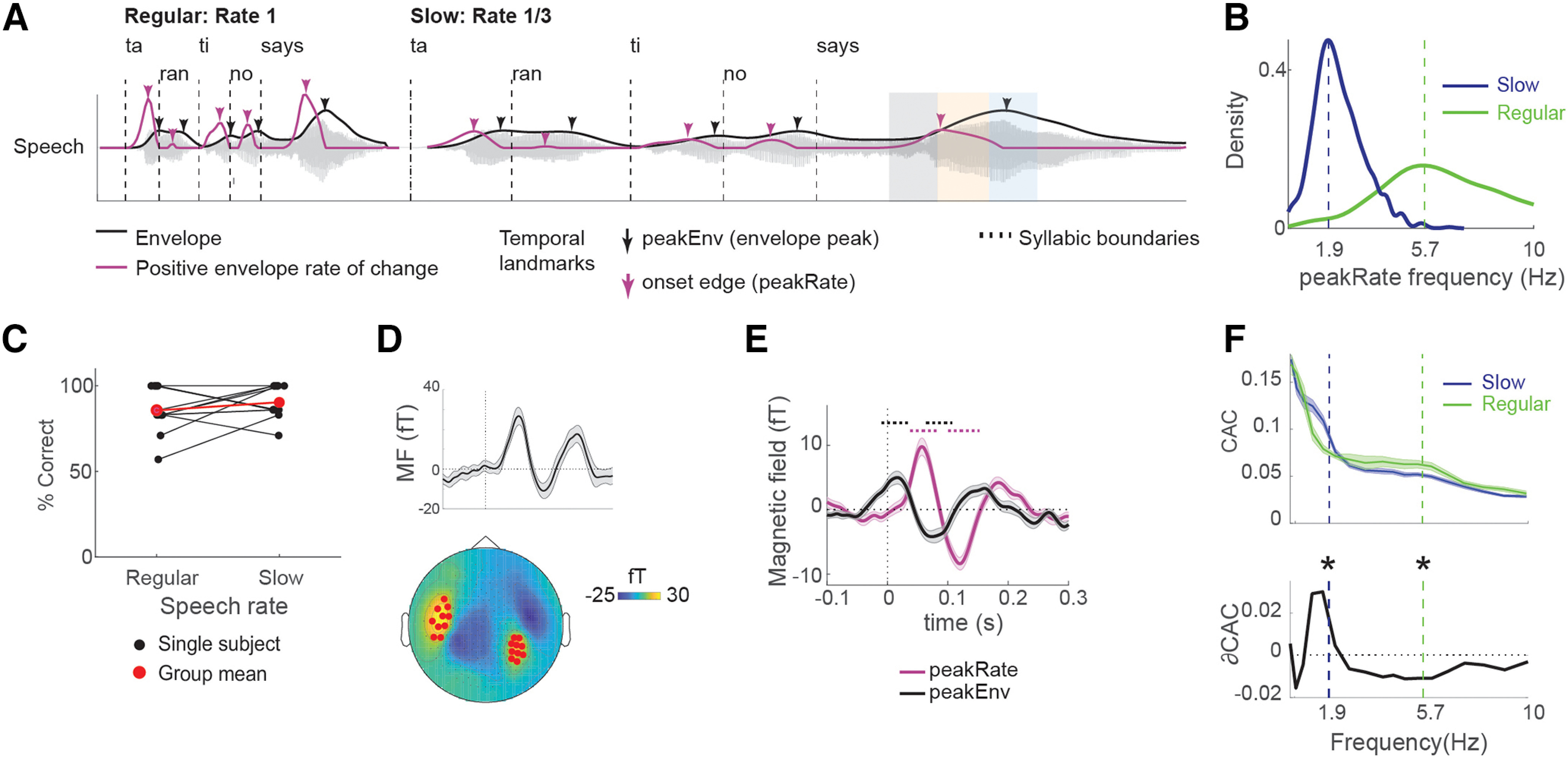
Task design and envelope tracking in neural data. ***A***, The acoustic waveform of an example utterance (“Tarantino says…”), with syllable boundaries, amplitude envelope, and first temporal derivative of the envelope superimposed on it. The same utterance is shown at a regular rate (left) and slowed (right) speech rate. Arrows indicate candidate temporal landmark that might induce phase-locking. Black represents local peaks in the envelope, peakEnv. Purple represents acoustic edges, defined as local peaks in the first temporal derivative (rate of change) of the envelope, peakRate. For transcripts of the entire speech stimulus, see Extended Data Table 1-1. For example stimulus excerpts at two different speech rates, see Extended Data 1 and Extended Data 2. ***B***, Frequency of occurrence for peakRate/peakEnv events. Dashed vertical lines indicate the average frequency of peakRate events in slow (blue, 1.9 Hz) and regular speech (green, 5.7 Hz). ***C***, Single-subject (black) and group-average (red) comprehension performance. For a list of all comprehension questions, see Extended Data Table 1-2. ***D***, Sensor selection was based on M100 response to utterance onsets. Top, Group-averaged evoked response across all 20 sensors included in the analysis. Error bars indicate ±1 SEM across subjects. Bottom, Topographic map of a group-averaged M100 response with selected sensors marked in red. ***E***, Group-averaged evoked response aligned to peakRate and peakEnv events. Dotted lines indicate clusters with *p* < 0.05 with a cluster-based permutation test against 0. Error bars indicate ±1 SEM across subjects. ***F***, CAC between MEG responses and speech envelope (top), and the difference between slow and regular speech (ΔCAC, bottom). Data were filtered in semi-logarithmically spaced bands between 0.3 and 10 Hz for this analysis. Dashed vertical lines indicate the average frequency of peakRate events in each condition, as shown in ***D***. *p < 0.01, *post hoc t* tests with interaction *p* < 0.01. Error bars indicate ±1 SEM across subjects.

We extracted the broad amplitude envelope of speech stimuli by applying rectification, low-pass filtering at 10 Hz, and downsampling to 100 Hz, to the original stimulus waveform (in this order). We then calculated the derivative of the resulting envelopes as a measure of its rate of change. Finally, we extracted the sparse time series of local peaks in the amplitude envelope (peakEnv) and its derivative (peakRate). All features are depicted in Figure 1*A*, for an example stimulus excerpt. Overall, the stimulus set contained 2106 peakRate and 2106 peakEnv events per speech rate condition.

### Evoked response and oscillatory entrainment models for interevent phase coherence (IEPC) simulation

We implemented two computational models that predict neural activity in response to continuous speech: one based on oscillatory entrainment and another based on evoked responses. We then submitted their output to the same phase analysis as for MEG data. We assumed that both processes were driven by peakRate events, based on our analysis of responses to acoustic landmarks and previous work (Oganian and Chang, 2019). As input, each model received a time series that contained peakRate values, scaled within speech rate between 0.5 and 1, at times of peakRate events, and zeros otherwise. We scaled to this range as our analyses revealed that neural phase alignment to speech is normalized within each speech rate, and that its magnitude for the bottom quantile is ∼50% of the top quantile (see Results; Fig. 5). To capture the variable latency of the neural response to nontransient sensory events, such as acoustic landmarks, we added random temporal jitter (Gaussian distribution, SD = 10 and 30 ms in regular and slow speech, respectively) to the time stamp of each peakRate event. Subsequent phase analyses were conducted using the original, nonjittered time stamps. To account for the nonuniform spectral impact of the 1/f noise that is typical to neurophysiological measurement, we added noise with this spectral content to the predicted neural response output by each model, with a signal-to-noise ratio of 1/10. To create the noise, we filtered Gaussian white noise to the 1/f shape with the MATLAB function firls.m. The temporal and amplitude jitter parameters were fitted to maximize the similarity between the predicted and observed spectro-temporal patterns of phase alignment. Importantly, to not favor one model, this was done across both models and speech rates. To ensure that results would not be biased by the introduction of simulated random noise, we repeated the randomization procedure 2560 times for each model and each speech rate (64 iterations of temporal noise × 40 iterations of amplitude noise), calculated the phase analyses (below) on the predicted neural signal from each randomization, and then averaged across randomizations.

For the oscillator model, peakRate events induce phase corrections of a fixed-frequency oscillator whose frequency is centered on the speech rate (5.7 and 1.9 Hz for regular and slow speech, respectively), as is assumed by oscillatory entrainment models and confirmed in previous work (Large and Snyder, 2009; Breska and Deouell, 2017). Following Large and Snyder (2009), this process was modeled using a coupled oscillator dynamical system as follows:

$$\frac{d\theta }{dt}=2\pi F-c\cdot \frac{s\left(t\right)}{r}\cdot sin\theta$$

$$\frac{dr}{dt}=r\left(1-r^{2}\right)+c\cdot s(t)\cdot cos\theta$$

**Figure 2.**
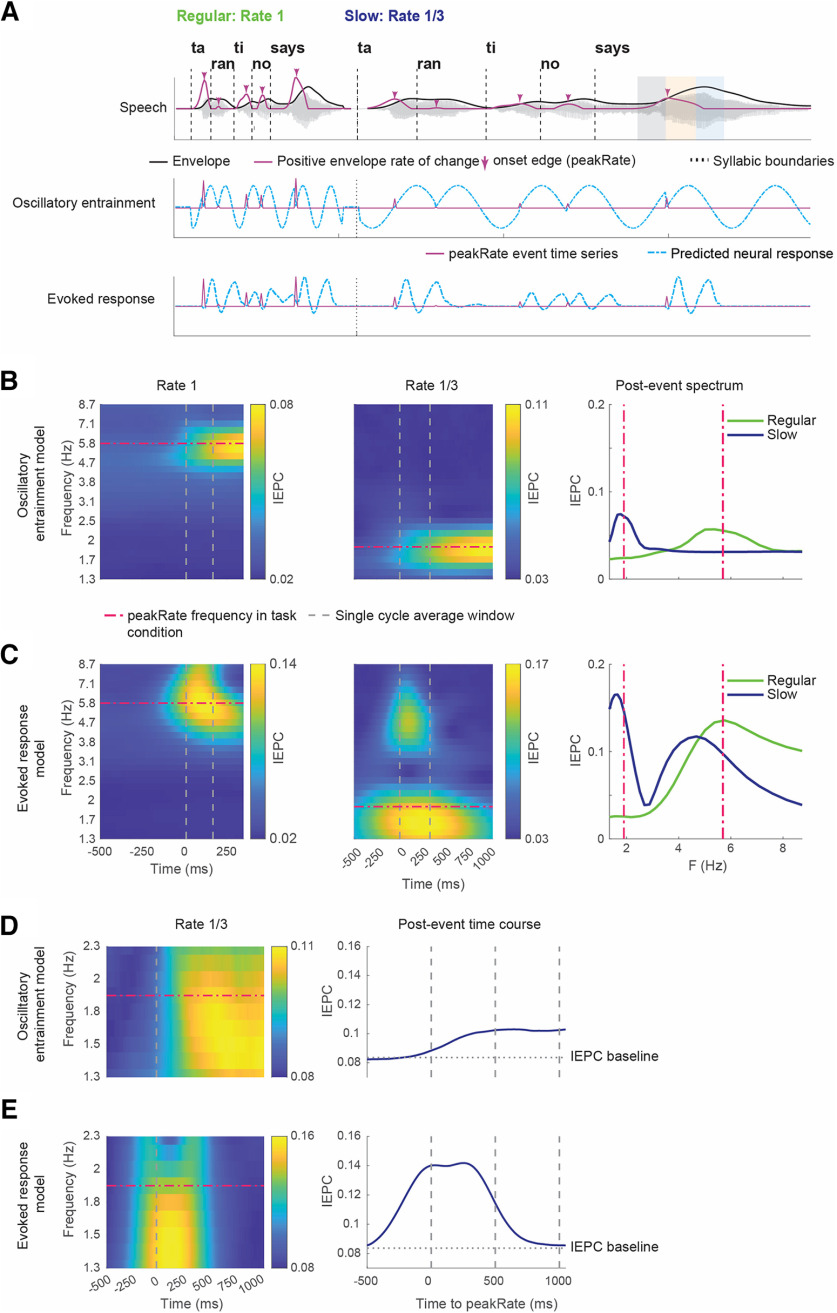
Spectral and temporal signatures of IEPC in oscillatory entrainment and evoked response models. ***A***, Schematic illustrations of the predicted neural response to the utterance in Figure 1*A* using three different models. Top, Speech signal. Middle, Oscillatory entrainment model. Bottom, Evoked response model. ***B***, IEPC patterns predicted by oscillatory entrainment model for regular and slow speech with a focus on spectral precision. Dashed lines indicate the frequency of peakRate events in each condition. ***C***, Same as in ***B***, but for evoked response model. ***D***, Temporal dynamics of IEPC in the delta frequency range predicted by oscillatory entrainment model, based on peakRate events that are at least 1000 ms apart from following events (*n* = 113 events) in the Slow speech condition. ***E***, Same as in ***D***, but for the evoked response model.

The system produces periodic limit cycle behavior at a radius of *r* = 1 (attractor point) and a frequency *F* in the absence of input (*s(t)* = 0) and follows phase correction toward an angle of θ=0 when presented with input (*s(t)* > 0). The magnitude of phase correction depends on the strength of the input, the current angle, and the coupling parameter *c*. At low values of *c*, no oscillator was able to entrain to speech, whereas at high values, entrainment spread across all oscillator frequencies. Crucially, as predicted, at intermediate values, only the oscillator with the correct frequency was entraining to our speech stimulus (see Fig. 2*B*). We thus focused on an oscillator model with intermediate entrainment strength and oscillator frequency corresponding to the speech rate in each task condition for further analyses. Specifically, the value of *c* was set such that the maximal phase correction possible (when *s(t)* = 1 and $\theta =\frac{\pi }{2}$ or $-\frac{\pi }{2}$) would be 70% of the maximal phase shift. We reconstructed the predicted response as follows: $PredResp_{i}=cos\theta_{i}\cdot r_{i}.$

peakRate events trigger a prototypical evoked response with its amplitude proportional to the strength of the input. For the evoked response model, this process was modeled using a linear convolution of the time series of peakRate events with the waveform of an evoked response to peakRate events. The latter was estimated directly from the MEG data, using a time-delayed linear encoding model (Temporal Receptive Field) (Holdgraf et al., 2017; Oganian and Chang, 2019), with a time window of −150 to 450 ms relative to peakRate events. While we found no effect of speech slowing on the shape of the neural response to peakRate events in our previous intracranial work (Oganian and Chang, 2019), we assumed that neural responses recorded with MEG will be additionally shaped by other speech features that occur in temporal proximity to peakRate events (e.g., vowel onsets), although our dataset did not allow us to explicitly model such additional features. Rather, we estimated the evoked response separately within each speech rate. We used the Temporal Receptive Field approach instead of simple averaging because of the high rate of peakRate events (average interval ∼170 ms), which would have distorted the averaging-based estimate because of overlap between evoked responses.

### MEG data preprocessing

Offline data preprocessing included (in this order) artifact rejection with dual signal subspace projection and downsampling to 400 Hz. Dual signal subspace projection is an MEG interference rejection algorithm based on spatial and temporal subspace definition (Sekihara et al., 2016). Its performance has been recently validated using clinical data (Cai et al., 2019). In all subsequent analyses of segmented data, segments containing single sensor data >1.5 pT and visually identified artifacts (including muscle, eye blink, and motion) were flagged as bad events and removed from further processing (0.2% of segments).

### Sensor selection

To focus analyses on responses originating in temporal auditory areas, we selected sensors based on the magnitude of the group-averaged M100 response to the onset of utterances (independent of responses to acoustic features within the utterance, which were the focus of subsequent analyses). For this purpose, we segmented the broadband signal around utterance onsets (−200 to 500 ms), averaged these epochs across utterances and participants, applied baseline correction (−200 ms to 0 ms relative to utterance onset), and extracted the M100 amplitude as the average activity between 60 and 100 ms after utterance onset. We then selected the 10 sensors with maximal M100 responses from each hemisphere. All subsequent analyses were conducted on these 20 sensors.

### Event-related analysis and sensor selection

For broadband-evoked response analysis, we first extracted the broadband signal by bandpass filtering the data between 1 and 40 Hz (second-order Butterworth filter).

To identify which landmark in the speech envelope drives evoked responses, we analyzed evoked responses to peakRate and peakEnv events. We reasoned that, with alignment to an incorrect landmark, evoked responses would have reduced magnitude because of smearing, and latency that is shifted away from the acoustic event. For this purpose, we segmented the broadband signal around acoustic landmark events (−100 to 300 ms), averaged these epochs across events within each participant separately for peakRate and peakEnv events, and applied baseline correction (−100 ms to 0 ms relative to event onset). Based on our previous work (Oganian and Chang, 2019), we hypothesized that peakRate events would be the driving acoustic landmark. We compared evoked responses to peakRate and peakEnv using time point by time point *t* tests.

### Time-frequency (TF) decomposition

Identical TF analyses were performed on the continuous MEG data and on the continuous simulated signal from the Evoked Response and Oscillatory Entrainment models. To evaluate the instantaneous phase of the signal at individual frequency bands (logarithmically spaced between 0.67 and 9 Hz, 0.1 octave steps), we applied noncausal bandpass Butterworth filters around each frequency of interest, performed the Hilbert transform, and obtained the amplitude and phase as the absolute value and phase angle, respectively, of the Hilbert signal. Filter order was chosen to achieve maximal 3 dB of passband ripple and at least 24 dB of stopband attenuation. We conducted this TF analysis with a narrow filter width (±0.1 octave of the frequency of interest) for analyses of spectral patterns to increase frequency resolution, and again with a wider filter (±0.5 octave) for analyses of temporal dynamics to increase temporal resolution.

### Cerebro-acoustic phase coherence (CAC)

To assess CAC between the speech envelope and MEG responses, the speech envelope was processed using the same procedure that was applied to the MEG responses: downsampling and TF analysis using the wide filter settings. Phase-locking between the speech envelope and MEG response was calculated across the entire duration of every utterance within each frequency band, using the CAC as follows:

$$CAC(\varphi )=\frac{1}{T}\left|\overset{T}{\underset{t=1}{\sum }}exp(i*(ph(\varphi ,t)-phs(\varphi ,t)))\right|$$

where φ is the center frequency of a frequency band, *T* is the number of time samples in an utterance, *ph* is the phase of the neural signal, and *phs* is the phase of the speech envelope in band φ at time *t*. To equate the number of time points entering the analysis for slow and regular speech, slow speech utterances were split into three equal parts before CAC calculation, and resultant CAC values were averaged. CAC was averaged across sensors for each hemisphere.

*A priori*, we hypothesized that CAC would differ between conditions in the frequency bands corresponding to the average frequency of peakRate events in each rate condition (regular: 5.7 Hz; slow: 1.9 Hz, see Fig. 1*B*). We tested this hypothesis using a three-way repeated-measures ANOVA with factors frequency band (high/low), factor speech rate (slow/regular), and hemisphere (left/right). To test for further differences in each frequency band, we assessed the effect of speech rate and hemisphere onto CAC using a two-way repeated-measures ANOVA with factor speech rate (slow/regular) and hemisphere (left/right). Significance in this analysis was Bonferroni-corrected for multiple comparisons across bands.

### IEPC

Both IEPC analyses were conducted on the actual MEG data and the neural responses predicted by the evoked response and oscillatory entrainment models. To assess neural phase-locking around peakRate events, we segmented the continuous phase data around peakRate events (see below), and obtained a time-resolved IEPC (Lachaux et al., 1999). For each time point, IEPC was calculated using the following formula:

$$IEPC(\varphi ,t)=\frac{1}{N}\left|\overset{N}{\underset{k=1}{\sum }}exp(i*ph_{k}(\varphi ,t))\right|$$

where *N* is the number of events, *ph* is the phase of the neural signal in trial *k*, for the frequency band φ and time point *t*. IEPC were first calculated within each of the selected sensors, then averaged across sensors.

#### Spectral patterns of IEPC

To assess the spectral distribution of phase-locking following peakRate events with increased frequency resolution, we segmented the phase data outputted by the narrow filter TF analysis around peakRate events (−500 to 500 ms) and calculated the IEPC. To prevent distortion of the estimated phase by subsequent peakRate events, we only used ones that were not followed by another peakRate event within the 0-500 ms window (*n* = 813 within each participant). To identify whether in this time window and frequency range there was a significant increase in IEPC in the MEG data, the resulting time × frequency IEPC was compared with the pre-event baseline using 2D cluster-based permutation *t* tests (Maris and Oostenveld, 2007) with 3000 permutations, a peak *t* threshold of *p* < 0.01, and a cluster threshold of *p* < 0.01. Baseline IEPC was calculated as the average IEPC between −400 and −100 ms relative to event onset in each frequency band.

To compare between model predictions and data, IEPC spectral profiles were calculated, separately for each speech rate condition, by averaging IEPC TF images following peakRate event onset across a time window that conforms to one cycle of an oscillator whose frequency matches the speech rate (i.e., 0-170 ms at regular speech rate and 0-500 ms at slowed speech rate).

#### Temporal extent of IEPC

To assess the temporal extent of IEPC between peakRate events, we focused on the slowed speech condition, where phase-locking originating from the evoked response and from putative oscillatory entrainment occupy distinct spectral bands. We segmented the phase data outputted by the broad filter TF analysis around peakRate events (−500 to 1000 ms) with a temporal interval of more than two oscillatory cycles for half an octave around the frequency of peakRate events (1.9 Hz): that is, at least 1040 ms to the next peakRate (*n* = 114 peakRate events per participant). As this analysis was focused on the temporal dynamics of IEPC, we examined IEPC dynamics as a function of time, averaged across single frequency bands in this range. For the MEG data, this time course was tested against a theoretical chance level, defined as the expected IEPC value for randomly sampling a matched number of angles from a uniform von Mises distribution.

### Effect of peakRate magnitude on IEPC

In each rate condition, peakRate events were split into five quantiles, and IEPC was separately calculated within each quantile. Then, we extracted the average IEPC in the theta band (4-8 Hz) across all the time points for one cycle of the given frequency band after the event. IEPC in each quantile was compared using two-way ANOVA with factors quantile and speech rate (regular speech, slow speech).

### Effect sizes and power

With over 1000 events (trials) per participant, our dataset is well powered beyond what is typically discussed in psycholinguistic studies, where the number of trials is mostly limited by stimulus selection (e.g., Brysbaert, 2019). For all comparisons, we report *post hoc* power analyses with effect sizes (dz) and beta power, calculated with the software G*power (Faul et al., 2009).

### Data and code availability

All custom-written analysis code will be publicly available on publication on github (https://github.com/ChangLabUcsf/MEG-SlowSpeech). Data will be made available on request from the corresponding authors.

## Results

### Speech envelope tracking for regular and slow speech as seen in MEG

We recorded MEG while participants (*n* = 12) listened to continuous speech containing 2106 instances of each envelope landmark, at the original rate (Regular speech condition 6.5 min duration), and once slowed to one-third of the original speech rate (Slow speech condition, 19.5 min duration, Fig. 1*A*). With this high number of events per condition, we were able to see clear and robust effects based on data from 12 participants (Stefanics et al., 2010) (for details on power calculation, see Materials and Methods). Stimuli were split into 26 utterances of 10-69 s duration (30-210 s in Slow speech condition), with additional silence periods inserted between them. This allowed us to estimate an auditory evoked response to speech onset from the data, without altering the original temporal dynamics of the stimulus within sentences.

In a first step, we characterized the temporal dynamics of acoustic landmark events in our speech stimulus, focusing on peaks in the rate of envelope change (peakRate, *n* = 2106 per condition, Fig. 1*A*) and on peaks in the envelope (peakEnv, *n* = 2106 per condition, black in Fig. 1*A*). In the regular speech condition, the average frequency of landmarks (similar for peakRate and peakEnv) was 5.7 Hz (SD = 2.9 Hz, Fig. 1*B*), as is typical in natural speech (Ding et al., 2016). In the slow speech condition, the average frequency of landmarks was 1.9 Hz (SD = 1 Hz, similar for peakRate and peakEnv), shifting the peak of the envelope power spectrum to the δ band. Slowing did not impair participants' comprehension, as probed by multiple choice comprehension questions after each story (3 or 4 questions per story, chance level per question: 50%; accuracy in regular speech: mean = 83%, SD = 13%; accuracy in slow speech: mean = 90%, SD = 9.5%; *t*_(11)_ = −1.85, *p* = 0.09; Fig. 1*C*).

### Acoustic edges drive MEG-evoked responses

We first asked which landmark in the speech envelope drives evoked responses and phase-locking to the envelope in regular speech. To focus our analyses on sensors that capture auditory sensory processing, we selected 10 sensors with the largest M100 response to speech onsets after silence periods from each hemisphere for all further analyses (Fig. 1*D*). The M100 response showed the typical dipole pattern in each hemisphere (Chait et al., 2004). First, we examined the characteristics of evoked responses (bandpass filtered 1-40 Hz and averaged in the time domain) locked to peakRate and peakEnv landmark events. While peakEnv closely follows on peakRate in regular speech, the interval between them varies. Thus, aligning to the incorrect landmark should lead to (1) a reduced magnitude of the averaged evoked neural signal because of smearing, and (2) shifts in response onset times away from the acoustic event. We found transient evoked responses with both alignments (Fig. 1*E*). Crucially, the evoked response was of larger magnitude when aligned to peakRate than to peakEnv (peak magnitude: *t*_(11)_ = 5.9, *p* < 0.001). Moreover, this response started after peakRate events, but before peakEnv events (response latency relative to the event for peakEnv: −12.5 ms; peakRate: 50 ms, determined as the first significant time point in a cluster-based permutation test against 0). Together, these analyses indicated that peakRate events, that is, acoustic edges, rather than peakEnv events, that is, envelope peaks, triggered the evoked response in MEG, in line with previous results (Gross et al., 2013; Doelling et al., 2014; Brodbeck et al., 2018; Oganian and Chang, 2019).

### CAC between speech envelope and MEG

To confirm that cortical speech envelope tracking was present in our data (Peelle and Davis, 2012), we calculated the CAC between neural responses and the speech envelope in frequency bands <10 Hz. CAC is typically increased at the frequency corresponding to the speech rate (Pefkou et al., 2017), which in our data corresponds to the frequency of peakRate in each rate condition (regular: 5.7 Hz, slow: 1.9 Hz). Indeed, speech rate had opposite effects on CAC in these two frequency bands (repeated-measures ANOVA, interaction *F*_(1,11)_ = 31.20, *p* < 0.001, η^2^ = 0.30, Fig. 1*F*). At 5.7 Hz, CAC was higher for regular speech (*t*_(11)_ = 5.6, *p* < 0.001, η^2^ = 0.42), while at 1.9 Hz it was higher for slow speech (*t*_(11)_ = 3.4, *p* = 0.006, η^2^ = 0.29). Moreover, CAC was overall higher at lower frequencies (*F*_(1,11)_ = 16.44, *p* < 0.001, η^2^ = 0.39), as is typical for this measure (Cohen, 2014). No other frequency band showed a significant effect of speech rate on CAC (all Bonferroni-corrected *p* > 0.05). Overall, this result replicates previous findings of cortical speech envelope tracking in frequency bands corresponding to the speech rate of the stimulus. However, as this measure is calculated across the entire stimulus time course, it cannot capture local temporal dynamics in the neural phase, driven by phase resets at acoustic edges. To evaluate local temporal and spectral patterns of neural phase-locking following peakRate events, we calculated IEPC across peakRate events in the speech stimulus. In contrast to prior studies of CAC, which quantified phase consistency across time, IEPC is calculated across single-event occurrences (i.e., single trials) for each time point. IEPC thus enables tracking of the temporal dynamics of phase-locking (Gross et al., 2013).

### Oscillator and evoked response models predict distinct patterns of phase alignment to slowed natural speech

To obtain a quantitative estimate of neural phase patterns predicted by oscillatory entrainment and evoked response mechanisms, we implemented computational models of neural envelope tracking as predicted by both processes (for a full description of both models, see Materials and Methods). The input to both models was the acoustic stimulus reduced to peakRate events: a continuous time series downsampled to match the MEG sampling frequency and containing non-zero values corresponding to peakRate magnitudes at times of peakRate events, and 0 otherwise. The oscillator model was implemented as a coupled oscillator dynamical system with a nondecaying amplitude attractor point, that followed phase resetting whenever the input was different from 0 (at peakRate events), at a magnitude determined by an entrainment parameter (Breska and Deouell, 2017). A preliminary analysis verified that indeed an oscillator whose endogenous frequency corresponds to the average rate of the speech stimulus would be best suited to entrain to the speech stimulus. The evoked response model was designed as a linear convolution of the peakRate event time series with a stereotypical evoked response, which was extracted from the actual MEG data using a time-lagged linear encoding model (rather than simulated to have an ideal shape) (Holdgraf et al., 2017; Oganian and Chang, 2019). To both models, we added 1/f shaped noise, as is observed in neurophysiological data, and a temporal jitter around peakRate event occurrence to each model (for a full description of both models, see Materials and Methods). Both models output a predicted neural response time series (Fig. 2*A*), from which we extracted predicted spectral and temporal patterns of IEPC in the theta-delta frequency ranges following peakRate events for each condition (Fig. 2*B*).

To identify distinct predictions of the two models, we focused on two aspects of the overall predicted pattern of IEPC. First, we quantified the spectral shape of predicted responses, by examining the average IEPC pattern in the first oscillatory cycle after peakRate events. We found that, in regular speech, both the evoked response model and the oscillatory model predicted a transient increase in theta IEPC following peakRate events (Fig. 2*B*,*C*, left). However, their predictions for the slow speech condition diverged significantly (Fig. 2*B*,*C*, middle). The oscillator model predicted a single peak in IEPC around the oscillator frequency in IEPC (Fig. 2*B*, right). In contrast, the evoked response model predicted two IEPC peaks, at ∼5.7 and ∼1.9 Hz, reflective of the shape of the evoked response (the higher frequency peak) and its frequency of occurrence (i.e., the frequency of peakRate events, the lower frequency peak), respectively (Fig. 2*C*, right). We verified this by manually morphing the shape of the evoked response and the frequency of evoked responses, which shifted the location of the upper and lower IEPC peaks, respectively.

Second, we examined the temporal extent of IEPC predicted by each model. A key feature of an oscillatory entrainment mechanism, which is central to the cognitive functions ascribed to oscillatory models, is that the endogenous oscillator will continue to reverberate after phase reset beyond the duration of a single oscillatory cycle, resulting in increased phase alignment for a prolonged time window (Haegens and Zion Golumbic, 2018; Helfrich et al., 2019; Meyer et al., 2020). In our data, this should be expressed as an increase in IEPC extending beyond a single oscillatory cycle after peakRate events. In contrast, if phase-locking is the result of evoked responses to peakRate events, the increase in IEPC should be limited to the duration of an evoked response. To quantify this, we focused our analysis on the first two cycles after peakRate events. To prevent interference from subsequent phase resetting events, we only included peakRate events that were not followed by another peakRate event in this interval (*n* = 114). Importantly, such events were distributed throughout the speech stimulus and not limited to sentence or phrase ends. As in regular speech rate, the duration of the evoked response (∼350 ms, Fig. 1*E*) extends across two putative cycles at the speech rate frequency (∼350 ms at 5.7 Hz), which would not allow to dissociate the two models; we focused this analysis on the slow speech condition. We then examined the time course of IEPC in a range of frequencies surrounding 1.9 Hz, the frequency of the putative oscillator that best entrains to the slow speech rate. As expected, we found divergent predictions: the oscillator model predicts that IEPC remains increased for multiple oscillatory cycles (Fig. 2*D*). In contrast, the evoked response model predicts that the increase in IEPC is temporally limited to the duration of a single evoked response (Fig. 2*E*). Together, this model comparison identified two divergent predictions for IEPC patterns in slow speech: the spectral distribution of IEPC and its temporal extent. Next, we performed these identical analyses on our neural data and compared the patterns in the data with the models' predictions.

### Spectral pattern of delta-theta phase-locking to acoustic edges is best described by the evoked response model

**Figure 3.**
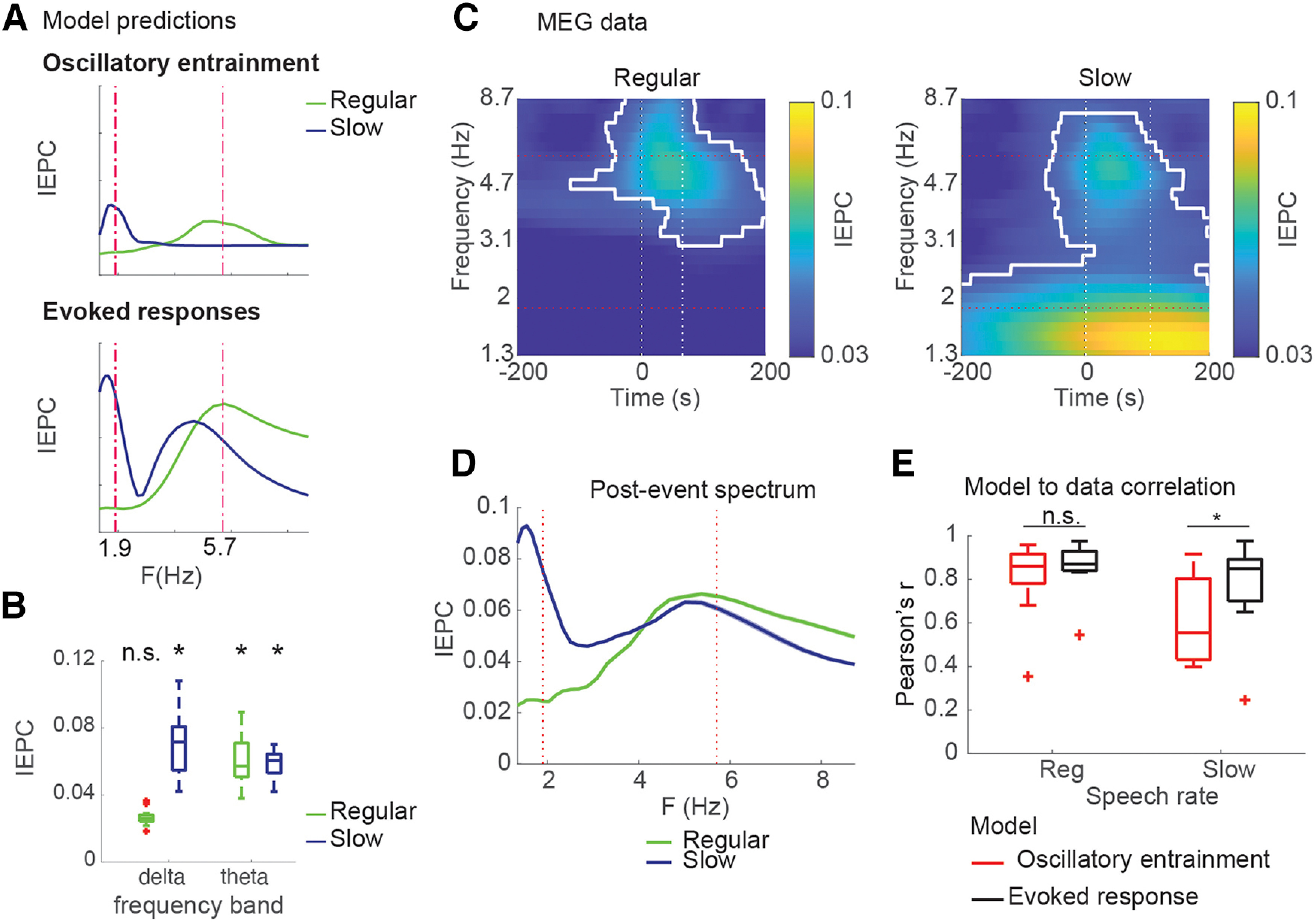
Spectral patterns of IEPC in MEG data. ***A***, Predictions of oscillatory and evoked response models for spectral distribution of phase-locking to peakRate events. ***B***, Average IEPC magnitudes observed in regular and slowed speech conditions within TF ROIs in theta and delta bands one oscillatory cycle after peakRate event. ***C***, IEPC patterns observed in MEG responses to speech at regular (left) and slowed (middle) rates. ***D***, Spectral IEPC profile averaged across time corresponds to predictions of the evoked response models (***A***, bottom). Significance contours in ***C***, ***D***, based on 2D cluster-based permutation testing against pre-event baseline (*p* < 0.001). ***E***, Correlation between IEPC time courses predicted by the models and observed in the neural data. *p < 0.05.

We next turned to testing the two divergent predictions of the two models against MEG data, starting with predictions for spectral distribution. Based on the models' predictions (Figs. 2, 3*A*), we first took a hypothesis-based approach, testing whether average IEPC values in predefined TF ROIs increased: within a single oscillatory cycle after peakRate event in the theta (4-8 Hz) and delta (1-3 Hz) ranges (Fig. 3*B*). In regular speech, we found significant IEPC increase (from theoretical baseline based on von Mises distribution) in the theta band (*t*_(11)_ = 6.9, *p* < 0.001, *d* = 2.1), but not the delta band (*p* > 0.5), consistent with both models (Fig. 3*A*). We then turned to the slow speech condition, where the predictions of the two models diverge. We found two spectral peaks in IEPC to peakRate events in slow speech, with a significant increase from baseline in the theta band (*t*_(11)_ = 8.5, *p* < 0.001, *d* = 3.1) and in the delta band (*t*_(11)_ = 5.2, *p* < 0.001, *d* = 1.9). This pattern is in line with the predictions of the evoked response model but not of the oscillator entrainment model (Fig. 3*A*), as the latter cannot explain the increased theta IEPC. To verify that these findings did not reflect the specific predefined TF ROIs, we complemented the ROI analysis with a data-driven 2D cluster-based permutation test. This analysis found one cluster in the theta band in the regular speech condition and a large cluster encompassing both theta and delta bands in the slowed speech condition (*p* < 0.001; Fig. 3*C*, white borders).

Finally, we directly compared how the predictions of both models fit with the spectral IEPC pattern in the data (see Fig. 3*D* for spectral patterns and Fig. 3*E* for model comparisons). As expected, the difference between models was not significant in the regular speech condition (oscillatory model: mean *r* = 0.86, evoked response model mean *r* = 0.81, *t*_(11)_ = 1.9, *p* = 0.06). Crucially, in the slowed speech condition, the evoked response model captured the IEPC dynamics significantly better than the oscillatory model (model comparison *t*_(11)_ = 3.8, *p* = 0.002), with a large effect size (*d* = 1.1, *post hoc* β = 0.93). This was because, while both models captured the delta-band peak in IEPC, only the evoked response model captured the IEPC dynamics in higher frequencies (oscillatory model: mean *r* = 0.46, evoked response model mean *r* = 0.7). Overall, the results of this analysis favor the evoked response model over the oscillatory model.

### Temporal extent of delta phase-locking is limited to a single cycle after peakRate events

**Figure 4.**
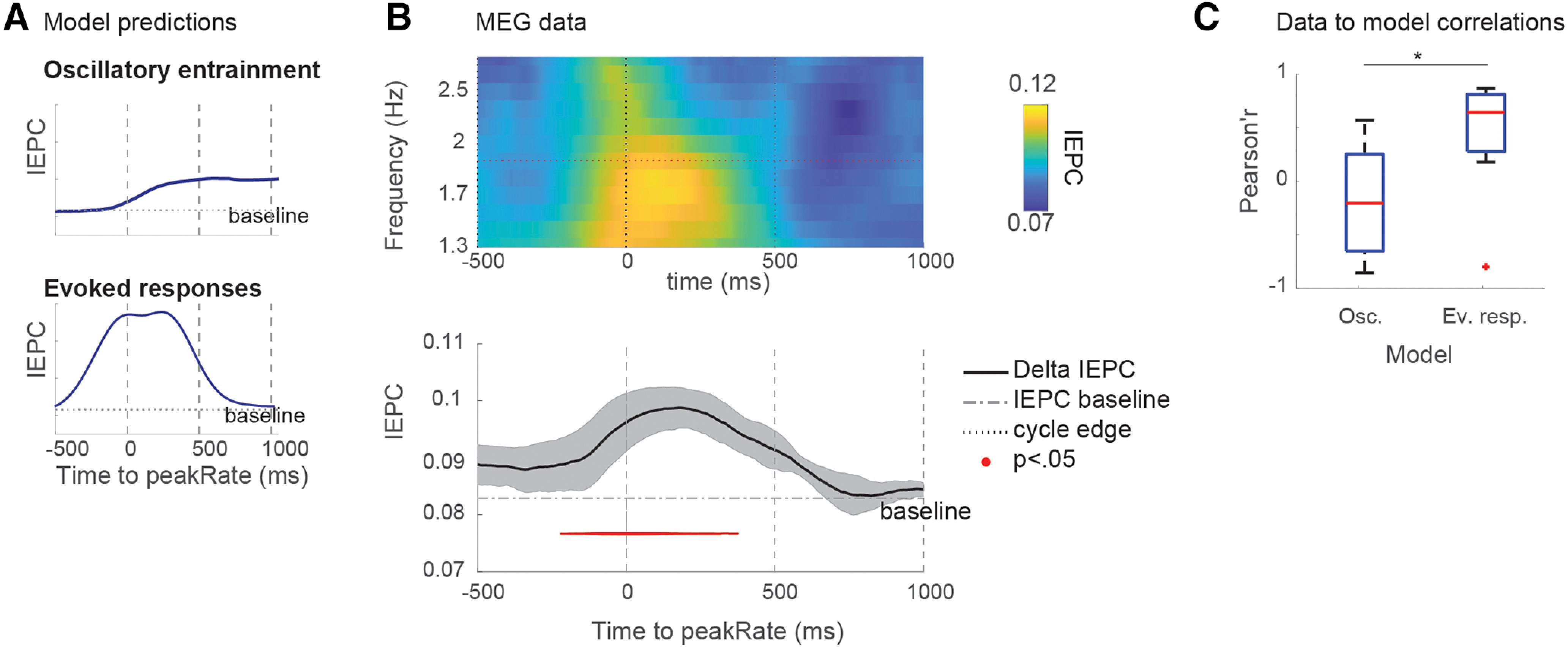
Delta phase-locking is limited to a single oscillatory cycle after peakRate events. ***A***, delta IEPC across selected peakRate events that were at least 200 ms away from preceding, and 1000 ms away from subsequent events. ***B***, delta IEPC time course. Bottom, IEPC average across the delta range. Red horizontal line indicates baseline. Red dots indicate time points of significant deviance from baseline. ***C***, Correlation between IEPC time courses predicted by the models and observed in the neural data. *p < 0.05.

We then examined the temporal extent of increased IEPC following peakRate events in the slowed speech condition. The oscillator model predicted that neural IEPC would remain elevated for at least oscillatory cycle, whereas the evoked response model predicted a transient increase in IEPC and return to baseline within 500 ms after the phase reset (Fig. 4*A*). We calculated IEPC for the MEG data on the same peakRate events as for the model simulations (duration of at least two cycles to subsequent peakRate events), which allowed us to test for continuous entrainment without interference by a subsequent event. We found that IEPC was elevated above baseline for a single cycle following peakRate events, but returned to baseline immediately after (Fig. 4*B*, cluster-based permutation test against theoretical baseline based on von Mises distribution). Notably, this pattern, including the latency of peak IEPC, closely followed the predictions of the evoked response model. Indeed, direct test of the fit of the models' predictions to the MEG data revealed strong significant correlation with the evoked response model (mean *r* = 0.59), but not with the oscillator model (mean *r* = −0.18). This was also reflected in a large significant effect in the direct comparison between models (*t*_(11)_ = 3.11, *p* = 0.009, effect size *d* = 0.9, *post hoc* power β = 0.8).

Finally, we explicitly tested in a hierarchical multiple regression model (data ∼ OSC model + ER model) whether the oscillatory model would explain variance in the data beyond the variance explained by the evoked response model. Second-level analyses on betas across participants showed a significant effect for the ER model (*t*_(11)_ = 3.34, *p* = 0.003), but no significant addition to the explained variance by the oscillatory entrainment model (*t*_(11)_ = −0.8, *p* = 0.2). This is in line with the negative correlation between data and the oscillatory model, which is because of the reduction in IEPC in the MEG data in the second oscillatory cycle, whereas IEPC remains high in the oscillatory model.

This analysis thus illustrates the transient nature of neural phase-locking to peakRate events, which is more consistent with an evoked response mechanism of speech envelope tracking, rather than with an oscillatory entrainment model. Collectively, our findings disagree with an oscillatory entrainment account, which postulates an oscillatory phase reset after an event, followed by continuous oscillatory reverberation. A more parsimonious account of our results is that the low-frequency phase-locking to the speech envelope in MEG is driven by evoked responses to peaks in the envelope rate of change (peakRate). Furthermore, our analysis shows that IEPC to peakRate events reflects the superposition of two different sources: (1) local responses to individual peakRate events and (2) the rate of occurrence of responses to peakRate events. Our analyses also demonstrate that the shift in IEPC frequency bands with changes in speech rate may be the product of a TF decomposition of a series of evoked responses, rather than a shift in the frequency of an entrained oscillator. This finding is a powerful illustration of the importance of explicit computational modeling of alternative neural mechanisms.

In the past, it has been suggested that evoked responses are reduced at slower speech rate, where peakRate magnitudes are smaller, limiting the usability of the evoked response model. In a final analysis, we thus tested whether IEPC to peakRate is normalized to account for changes in speech envelope dynamics induced by changes in speech rate.

### Speech rate normalization of peakRate IEPC

**Figure 5.**
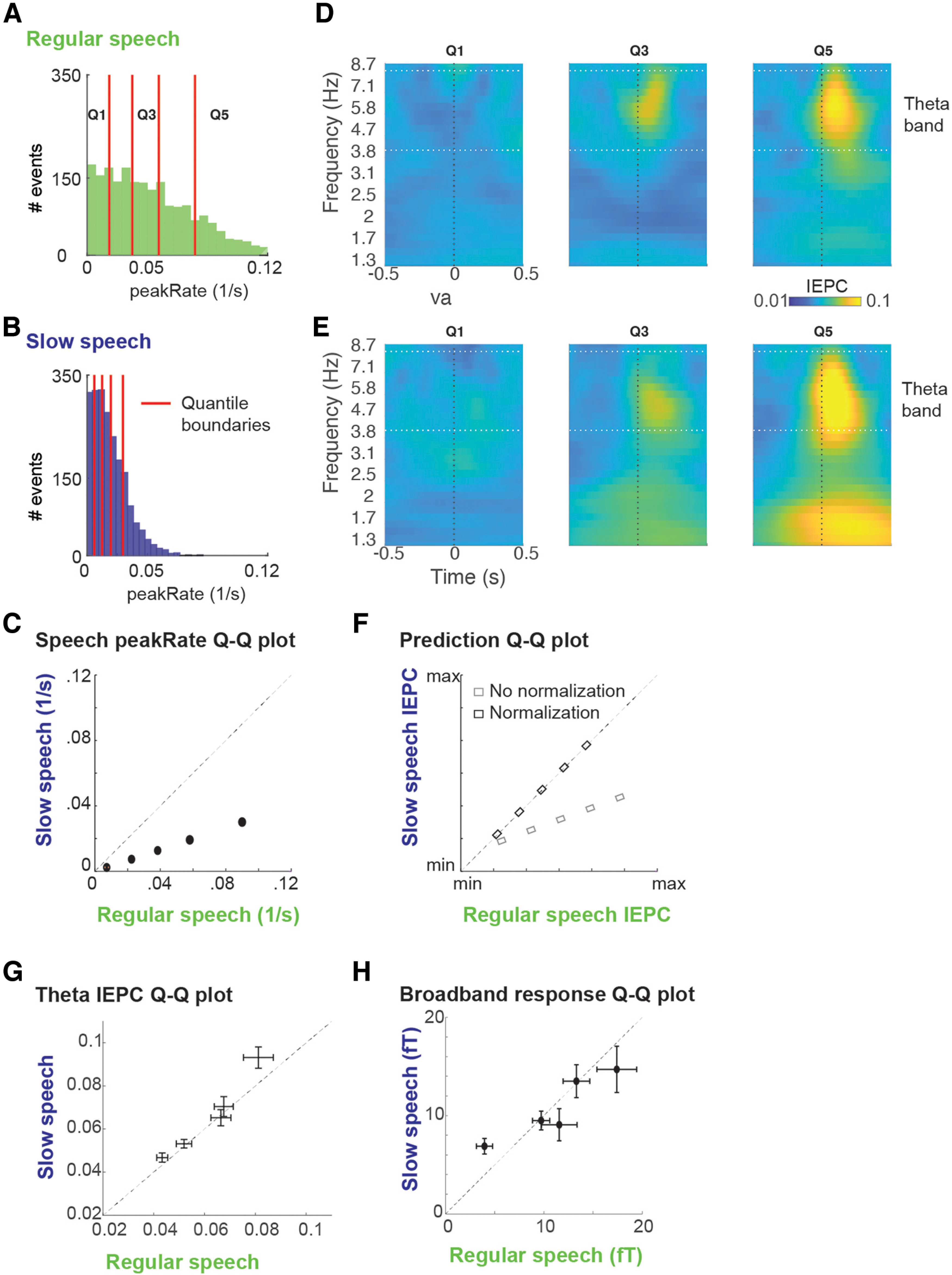
Normalization of peakRate IEPC for contextual speech rate. ***A***, Histogram of peakRate magnitudes in regular speech, with quantile boundaries marked in red. ***B***, Same as in ***A***, but for slow speech. ***C***, Quantile-quantile plot of peakRate magnitudes in regular and slowed speech stimulus. peakRate values in slowed speech stimulus are one-third of peakRate values in regular speech stimulus. ***D***, IEPC in first, third, and fifth peakRate magnitude quantile. Horizontal lines indicate the theta frequency range (4-8 Hz). ***E***, Same as in ***D***, but for slow speech. ***F***, Predicted quantile-quantile plots of theta IEPC in regular and slowed speech with (dark) or without (light) normalization. ***G***, Quantile-quantile plot of theta-band IEPC (mean; error bars indicate ±1 SEM across subjects) in regular and slow speech. Theta IEPC quantile-quantile values are close to the diagonal, indicating similar magnitudes of theta IEPC in regular and slowed speech conditions. ***H***, Quantile-quantile plot of broadband-evoked response peak magnitudes (mean, error bars indicate ±1 SEM across subjects) in regular and slow speech. Quantile-quantile values are close to the diagonal, indicating similar magnitudes of the broadband-evoked response to peakRate events in regular and slowed speech conditions.

The perceptual ability to adapt to variation in the speech signal resulting from changes in the speech rate (i.e., the number of syllables produced per second) is referred to as speech rate normalization. Changes in speech rate results in acoustic changes in the speech signal, including slower amplitude increases at acoustic edges, that is, lower peakRate magnitudes (Fig. 5*A*,*B*). We had previously found that responses to peakRate monotonically scale with peakRate magnitude, being larger for faster changes in the speech amplitude (Oganian and Chang, 2019). Efficient envelope tracking across speech rates would thus require remapping of neural responses to peakRate magnitude, to account for this overall reduction. Here, we assessed the effect of speech rate on the magnitude of theta IEPC to peakRate events. In the slowed speech, stimuli peakRate magnitudes were one-third of those in regular speech (Fig. 5*C*). If no normalization occurs, IEPC magnitudes in slow speech should reflect absolute peakRate values, resulting in an overall reduction in IEPC (Fig. 5*F*, dark dots). In contrast, if theta IEPC to peakRate is invariant to speech rate, it should reflect peakRate values relative to the contextual speech rate, resulting in similar IEPC magnitudes in both speech rate conditions (Fig. 5*F*, light dots).

An evaluation of IEPC after peakRate events, split by peakRate magnitude quantiles, showed comparable theta IEPC in both speech rate conditions (Fig. 5*D*,*E*), such that average theta IEPC was more robust for larger peakRate magnitudes across both rate conditions (the main effect of peakRate quantile: *b* = 0.01, SD = 0.001, *t* = 1.4, χ^2^ = 55.0, *p* = 10^−13^). Crucially, they did not differ between regular and slow speech (Interaction effect: *b* = 0.003, SD = 0.005, *t* = 0.6, not significant, Fig. 5*G*), as expected in case of speech rate normalization (Fig. 5*F*, dark dots). The same pattern was observed for the magnitude of peak evoked responses (Fig. 5*H*). Thus, the magnitude of phase reset induced by peakRate depended on its magnitude relative to the local speech rate context, allowing for the flexible encoding of peakRate information at different speech rates.

### Evoked low-frequency power following peakRate events

Evoked increase in power is a marker of evoked neural responses and is used to distinguish between evoked responses and oscillatory activity. In addition to calculating the ERP to peakRate events, we thus also tested whether band-passed power would increase after peakRate events. However, we found no significant effects of peakRate on evoked power in theta or delta bands (*p* > 0.05, cluster-based permutation test, data not shown). Our hypothesis that this was because of higher susceptibility of power measures to noise was confirmed in a simulation of the evoked response model (see below).

**Figure 6.**
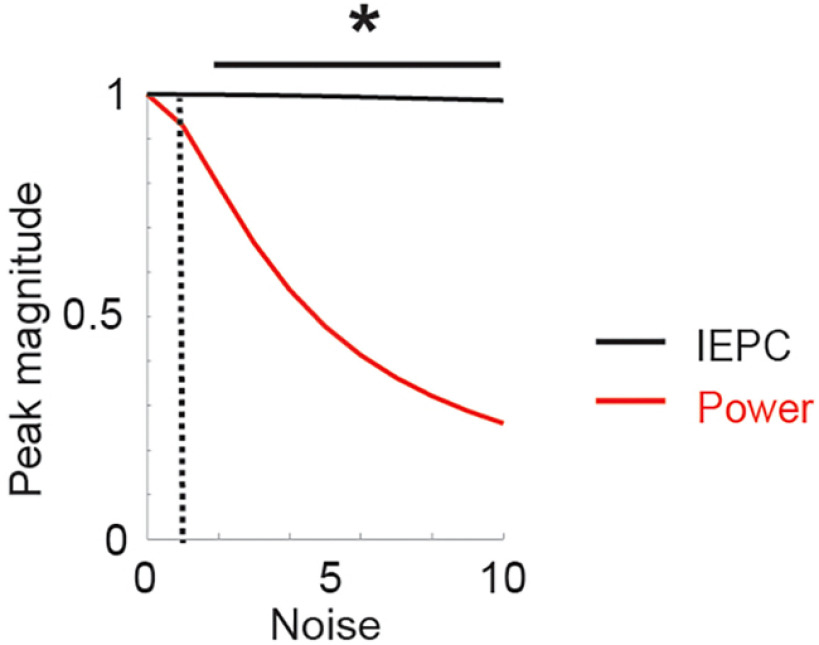
Effect of noise level on IEPC (black) and power (red) after peakRate events in theta band (4-8 Hz) for regular speech. *p < 0.01.

We hypothesized that this lack of increase in power in theta or delta bands following peakRate events might reflect the high susceptibility of power increases to noise. To assess the effect of noise onto power and phase measures, we tested the evoked response model at noise levels of 1-10 relative to response magnitude. We evaluated the effect of noise onto power and IEPC in the theta band (4-8 Hz) in the window of a single cycle for a given frequency band after event onset. The effects of noise on power and IEPC were compared using two-sided paired *t* tests at each noise level (*n* = 20 simulated responses), with Bonferroni correction for the number of comparisons. As predicted, we found continuously large effect sizes for IEPC even at high levels of noise, whereas the effect size for power deteriorated rapidly with the addition of noise (Fig. 6).

## Discussion

We evaluated local temporal dynamics in MEG neural representation of the continuous speech envelope against the predictions of oscillatory entrainment and evoked response models, derived from explicit computational models of both processes. In line with previous work, we found that acoustic edges (peakRate events) drove evoked responses and phase-locking over auditory cortical areas (Hertrich et al., 2012; Brodbeck et al., 2018; Oganian and Chang, 2019). Critically, however, only the evoked response model captured the spectral and temporal extent of phase-locking to acoustic edges: a transient local component in the theta range, reflective of the evoked response, and, spectrally distinct in slow speech, a separate global component, which captured the frequency of acoustic edges in the stimulus. An analysis of temporally sparse acoustic events further supported the evoked response model: phase-locking was transient and limited to the duration of the evoked response. This contradicts the pattern predicted by entrainment models, namely, sustained oscillatory phase-locking at the speech rate (Peelle and Davis, 2012; Helfrich et al., 2019). Finally, we found that the magnitude of the evoked phase reset to acoustic edges reflected the speech-rate-normalized amplitude slope at the acoustic edge, offering novel evidence for speech rate normalization. Our results establish acoustic edges as the basis for the representation of the speech envelope across methodologies and provide additional support against the representation of envelope peaks in the human speech cortex. Overall, our findings suggest that neural phase-locking induced by evoked responses to acoustic edges is the primary source of speech envelope tracking in the theta-delta band.

Neural phase resetting may be fully explained by the superposition of evoked responses or additionally also contain the entrainment of endogenous oscillatory activity. To distinguish between neural responses reflective of each, we derived the spectral and temporal patterns of phase-locking to acoustic edges using simulations of both mechanisms. Model predictions diverged in the slowed speech condition: Spectrally, the evoked response model predicted two spectral peaks in phase reset, in both theta and δ ranges, whereas oscillatory models predicted δ phase-locking only. Temporally, the evoked response model predicted only transient phase-locking at the speech rate, whereas oscillatory entrainment predicted reverberation: a persisting oscillation for at least 2 cycles after phase reset (Helfrich et al., 2019). The precise temporal extent of IEPC in the oscillator model depends on the decay parameter. However, the hallmark prediction of oscillatory models is that phase-locking will continue after phase reset beyond a single oscillatory cycle, which is the minimal temporal extent that allows for the model's proposed functional benefits. It was thus not necessary to include a decay parameter in our models.

In our data, both spectral and temporal patterns of phase-locking favored the evoked response model: two spectral peaks and temporally transient phase-locking. Notably, both models generated the low-frequency phase-locking component in the slow speech condition, corresponding to the frequency of acoustic edge events. While previous work interpreted this component in favor of oscillatory entrainment, our results show that only its temporal extent distinguishes between the two models (van Bree et al., 2022). Overall, our analyses show that a linear convolution of evoked responses to discrete acoustic edge events in speech is sufficient to account for the pattern of neural phase-locking to continuous speech. This finding has major implications for theories of speech perception. For instance, instead of oscillatory resonance, predictive processing of speech could rely on nonoscillatory temporal prediction mechanisms guided by statistical learning (Sohoglu and Davis, 2016; Friston et al., 2021).

Speech rate normalization is a central behavioral (Wade and Holt, 2005; Reinisch, 2016) and neural phenomenon in speech perception. Shifting of the entrained oscillatory frequency to match the input speech rate was previously proposed as its neural mechanism (Alexandrou et al., 2018b; Kösem et al., 2018). Here, however, we find that the shift of neural phase-locking to lower frequencies with speech slowing is an epiphenomenon of spectral analysis of a series of evoked responses. Instead, the magnitude of phase-locking to acoustic edges was normalized relative to the distribution of peakRate magnitudes at each rate. Namely, phase-locking was comparable across speech rates, despite flatter acoustic edges in slow speech. This suggests that the cortical representations of acoustic edges reflect the magnitude of an edge relative to the contextual speech rate. Such shifting of the dynamic range for acoustic edge magnitudes constitutes a flexible mechanism that maximizes the sensitivity to speech temporal dynamics (Diehl et al., 1980; Hirataa and Lambacher, 2004) and might not be limited to speech sounds.

Our approach represents a methodological departure from previous investigations of speech envelope tracking. Namely, previous studies focused on CAC, which reflects the consistency of phase differences between the neural signal and the acoustic stimulus across time (Peelle et al., 2013). CAC is primarily sensitive to regularities across time, such as the rate of phase resets. In contrast, we used IEPC, which focuses on assessing temporally local similarities in neural phase across repeated occurrences of the same acoustic event (for IEPC to speech onsets, see Gross et al., 2013). Our approach revealed that both local phase resets and their rate of occurrence are reflected in IEPC to acoustic edges. In regular speech, both components overlapped, whereas slowing of the speech signal revealed their distinct sources.

Speech rate manipulations are frequently used to study speech envelope tracking (Ahissar et al., 2001; Ghitza and Greenberg, 2009; Nourski et al., 2009; Pefkou et al., 2017). Most previous studies used compressed speech to study temporal boundaries on envelope tracking and intelligibility. In contrast, here we used slowed speech to spread distinct acoustic envelope features out in time. Notably, our approach required us to slow the speech signal by a factor of 3, which is rarely encountered in natural speech, except in clinical populations (e.g., subcortical degeneration), where speech can get very slow (Volkmann et al., 1992). Crucially as our participants adapted to the slow speech immediately, it is likely that our stimulus relies on the same perceptual mechanisms that are at play in the regular speech condition. This is also supported by our intracranial work, where responses to acoustic edges in slow (up to slowing factor of 4) and regular speech were qualitatively identical (Oganian and Chang, 2019). It is essential to reconsider previous findings under the evoked response framework. For example, while envelope tracking and intelligibility deteriorate for speech rates >8 Hz, insertion of brief silence periods in compressed speech, which returns the effective speech rate to <8 Hz, improves intelligibility (Ghitza and Greenberg, 2009). While this result is typically interpreted as evidence for oscillatory envelope tracking in the theta range, within an evoked response framework, it might be reflective of the minimal refractory period of neural populations that encode acoustic edges in speech.

Natural speech does not have a robust temporal rhythmicity (Alexandrou et al., 2018a). Our focus on envelope tracking for natural speech indicates that, in this case, neural signatures of envelope tracking are well explained by an evoked response model without the need for an oscillatory component. These results seemingly contradict recent findings of predictive entrainment to music (Doelling et al., 2019). However, our study used natural speech with considerable variability in interedge intervals, unlike in rhythmic musical stimuli. Critically, recent neuropsychological work dissociated neural mechanisms for prediction based on rhythmic streams from predictions in nonrhythmic streams (Breska and Ivry, 2018). This adds an important caveat to the current debate, suggesting that previous results may perhaps not extend to natural speech with inherent temporal variability and reduced rhythmicity. The present study thus calls to reevaluate the role of oscillatory entrainment in natural speech comprehension. However, it does not preclude the possibility that the introduction of additional rhythmicity to speech (e.g., in poetry or song) or occasionally more temporally regular everyday speech, particularly in longer utterances, recruits additional neural processes associated with the processing of rhythms.

Such additional processes might support speech comprehension and could underlie some of the recent findings obtained with a rhythmic speech stimulus (ten Oever and Sack, 2015; Ding et al., 2016; Zoefel et al., 2020). On the other hand, while intelligibility and phase patterns are affected by increased speech rhythmicity or concurrent rhythmic brain stimulation, such findings indicate that oscillations may enhance speech processing, but not that they are necessary for the representation of the significantly less periodic natural speech. Therefore, caution needs to be exercised when extending findings from rhythmic stimuli (e.g., Ding et al., 2016; Zoefel et al., 2018a; Doelling et al., 2019) to natural speech.

Overall, our results show that an evoked response model accounts for the main neural signatures of speech envelope tracking in MEG. This neural representation of acoustic edges informs about speech rate via interevent intervals. Moreover, the speech rate normalization of these responses renders this mechanism flexibly adaptable to changes in speech rate. Thus, evoked responses to acoustic edges track the syllabic rate in speech and provide a flexible framework for temporal analysis and prediction during speech perception.
