# Supplementary material for: Phase Alignment of Low-Frequency Neural Activity to the Amplitude Envelope of Speech Reflects Evoked Responses to Acoustic Edges, Not Oscillatory Entrainment
Source: J Neurosci. 2023 May 24;43(21):3909–21. doi: 10.1523/JNEUROSCI.1663-22.2023 (PMC10218004; doi:10.1523/JNEUROSCI.1663-22.2023)
Supplement: Table 1-1 — Speech stimulus transcription. Download Table 1-1, DOCX file. [file ns-JN-RM-1663-22-s03.docx]

**Table 1-1. Speech stimulus transcription**

| **Stimulus ID** | **Transcript** |
| --- | --- |
| f1ajrlp1 | Wanted: Chief Justice of the Massachusetts Supreme Court. In April, the S.J.C.'s current leader Edward Hennessy reaches the mandatory retirement age of seventy, and a successor is expected to be named in March. It may be the most important appointment Governor Michael Dukakis makes during the remainder of his administration and one of the toughest. As WBUR's Margo Melnicove reports, Hennessy will be a hard act to follow. |
| f1ajrlp2 | In nineteen seventy-six, Democratic Governor Michael Dukakis fulfilled a campaign promise to de-politicize judicial appointments. He named Republican Edward Hennessy to head the State Supreme Judicial Court. For Hennessy, it was another step along a distinguished career that began as a trial lawyer and led to an appointment as associate Supreme Court Justice in nineteen seventy- one. That year Thomas Maffy, now president of the Massachusetts Bar Association, was Hennessy's law clerk. |
| f1ajrlp3 | The author of more than eight hundred State Supreme Court opinions, Hennessy is widely respected for his legal scholarship and his administrative abilities. Admirers give Hennessy much of the credit for sweeping court reform that began a decade ago, and for last year's legislative approval of thirty-five new judgeships and three hundred million dollars to restore crumbling court houses. Despite the state's massive budget deficit, Hennessy recently urged colleagues in the bar association not to retreat from these hard won gains. |
| f1ajrlp4 | Hennessy is the S.J.C.'s thirty-second chief justice. Holding the court system on the course he has set and plotting it's future agenda won't be an easy job for his successor. |
| f1ajrlp5 | Attorney Haskel Kassler chairs the Judicial Nominating Council, eighteen attorneys and laypeople charged with screening applicants for vacancies on the bench. Usually the J.N.C. refers three nominees to the Governor. His top choice is rated by bar associations and grilled by the Governor's executive council. Kassler says, unlike the Federal Supreme Court, there's no litmus test on particular issues that Massachusetts high court nominees must pass. |
| f1ajrlp6 | All but one of the Chief Justices since eighteen ninety- nine, when Oliver Wendell Holmes was appointed, came from the ranks of S.J.C. associate justices. If he sticks with tradition, Dukakis is likely to elevate one of his appointees to chief. That means Paul Leocos or Ruth Abrams, the only woman on the court. Another possible choice is Herbert Wilkins, a Governor Sargent appointee, and next to Hennessy, the court's most senior member. The other three associate justices were put on the bench by Governor Edward King. And many lawyers say that despite Dukakis' promise to keep the judiciary above the political fray, it's unlikely Dukakis will choose a King appointee to run the state's highest court. For WBUR, I'm Margo Melnicove. |
| m2brrlp1 | Massachusetts may now have the toughest drunken driving law in the nation, thanks to the Safe Roads Act that became law this week. The legislation came complete with a message from the governor to those seeking a little too much comfort and joy this holiday season and beyond. |
| m2brrlp2 | Under the new law the consequences of driving drunk will be swift and severe. Under the old, already tough law, first time offenders used to lose their license for thirty days. Well now they could lose it for as many as ninety. The prison sentence for a second time offender has now doubled. The law is also tougher for vehicular homicide, and it cracks down on minors who drink and drive. State trooper Joseph Holly says this tough new law will mean fewer drunken drivers. |
| m2brrlp3 | Well that's what supporters of the Safe Roads Act are hoping anyway, but will it work? Massachusetts got tough on drunken drivers years ago. What's going to make the difference this time around? Ralph Henksen is chief of the social and behavioral science section at the Boston University school of public health. He says research into what deters the drunken driver shows that often those who drink and drive are risk takers. Even when they're sober they're more likely to run red lights and shun the seat belt. If they think they can drink and drive and get away with it, they'll try. |
| m2brrlp4 | And in fact the state is not planning on putting more police on the road. State officials say there will be the same levels of enforcement. B.U.'s professor Henksen believes the key to the new law's success is its enforcement. |
| m2brrlp5 | The state Safe Roads Act is only a few days old, but it's already facing a challenge. The man who directed the successful effort last election to get the mandatory seat belt law stripped from the law books is now vowing to try to repeal part of this law. Lynn activist Chip Ford has yet to give a name to his committee, but he's already set its goal: to repeal what is the most controversial clause in the Safe Roads Act, the per se clause, which allows someone to be punished for drinking and driving before they've even been tried in court. Under the per se part of this new law, if you're arrested, take a breath test, and your blood content level registers point one oh or higher, you can automatically lose your license for ninety days. And that's just at your arraignment. Then you still got to face DWI charges at a trial. State officials say about a quarter of all those arrested for drunken driving are repeat offenders. They say the per se clause will get those people off the road sooner. Chip Ford says it may indeed get them off the road, but it will do it at the expense of their civil rights. |
| m2brrlp6 | Ford and other opponents of the per se clause say it relies exclusively on the breath test, and they say breathalizers are simply unreliable. John Tarantino is a Providence attorney who specializes in drunken driving cases. |
| m2brrlp7 | Tarantino says because of people's physiological differences, the breathalizer test will always be inaccurate on about fourteen percent of the population. Those people will register as having more alcohol in their blood than they actually do. But state officials aren't convinced that's a problem. While they don't say breathalizers are one hundred percent accurate, they do say they are extremely reliable, and if they err at all it's on the conservative side, the side favorable to the driver. As for those who say that taking away someone's license based on the breathalizer results is tantamount to trial by machine, state officials say that simply isn't so. Losing one's license under the per se clause is not a criminal offense. You won't get a criminal record for it. It is a civil infraction and one that they believe will be a key deterrent to the drunken driver. If the per se clause is tested in court, the state says it will pass constitutional muster. And if opponents gather enough signatures to make it a ballot question in nineteen eighty-eight, state officials predict voters will keep it intact. |
