# Supplementary material for: Phase Alignment of Low-Frequency Neural Activity to the Amplitude Envelope of Speech Reflects Evoked Responses to Acoustic Edges, Not Oscillatory Entrainment
Source: J Neurosci. 2023 May 24;43(21):3909–21. doi: 10.1523/JNEUROSCI.1663-22.2023 (PMC10218004; doi:10.1523/JNEUROSCI.1663-22.2023)
Supplement: Table 1-2 — Comprehension questions. Download Table 1-2, DOCX file. [file ns-JN-RM-1663-22-s04.docx]

**Table 1-2. Comprehension questions**

| **Stimulus ID** | **Question** | **Answer** |
| --- | --- | --- |
| f1ajrlp1 | What is the mandatory retirement age for chief justice? | **a: 70** b: 30 |
| f1ajrlp2 | What did Michael Dukakis promise in his campaign? | a: To legalize medical Marijuana, **b: To de-politicize judicial appointments.** |
| f1ajrlp3 | How many laws were passed under Hennessy? | **a: more than 800** b: more than 8000 |
| f1ajrlp4 | How many chief justices were there before Hennessy? | **a: 31** b: 10 |
| f1ajrlp5 | How many nominees for the court are passed to the governor? | a: 5 **b: 3** |
| f1ajrlp6 | How many chief Justices so far were not from the ranks of SJC associate justices? | a: 5 **b: 1** |
| m2brrlp1 | What is the name of the new law in Massachusetts? | **a: Safe Roads Act**, b: Seatbelt act |
| m2brrlp2 | For how long do first-time offenders lose their license under the new law? | a: 10 days, **b: 90 days** |
| m2brrlp3 | Research shows that those that drink, and drive are | **a: risk-takers**; b: not aware of the risks |
| m2brrlp4 | Are there more police on the road after the new law? | a: Yes, **b: No** |
| m2brrlp5 | How many of those arrested for drunk driving are repeat offenders? | **a: a quarter** b: More than half |
| m2brrlp6 | Is the breathalyzer test reliable? | a: yes, **b: no** |
| m2brrlp7 | Does one get a criminal record for losing their driver’s license? | a: yes, **b: no** |
